# COVID-19 the showdown for mass casualty preparedness and management: the Cassandra Syndrome

**DOI:** 10.1186/s13017-020-00304-5

**Published:** 2020-04-09

**Authors:** Federico Coccolini, Massimo Sartelli, Yoram Kluger, Emmanouil Pikoulis, Evika Karamagioli, Ernest E. Moore, Walter L. Biffl, Andrew Peitzman, Andreas Hecker, Mircea Chirica, Dimitrios Damaskos, Carlos Ordonez, Felipe Vega, Gustavo P. Fraga, Massimo Chiarugi, Salomone Di Saverio, Andrew W. Kirkpatrick, Fikri Abu-Zidan, Alain Chicom Mefire, Ari Leppaniemi, Vladimir Khokha, Boris Sakakushev, Rodolfo Catena, Raul Coimbra, Luca Ansaloni, Davide Corbella, Fausto Catena

**Affiliations:** 1 General, Emergency and Trauma Surgery Department, Pisa University Hospital, Via Paradisia 1, 56100 Pisa, Italy; 2 General and Emergency Surgery, Macerata Hospital, Macerata, Italy; 3 Division of General Surgery, Rambam Health Care Campus, Haifa, Israel; 4 3rd Department of Surgery, Attiko Hospital, MSc “Global Health-Disaster Medicine”, National and Kapodistrian University of Athens (NKUA), Athens, Greece; 5 MSc “Global Health-Disaster Medicine” School of Medicine, National and Kapodistrian University of Athens (NKUA), Athens, Greece; 6 Ernest E Moore Shock Trauma Center, Denver Health, Denver, CO USA; 7 Trauma Surgery Department, Scripps Memorial Hospital, La Jolla, San Diego, CA USA; 8 Surgery Department, University of Pittsburgh, Pittsburgh, PA USA; 9 Deparment of General & Thoracic Surgery, University Hospital of Giessen, Giessen, Germany; 10 Chirurgie Digestive, CHUGA-CHU Grenoble Alpes, Grenoble, France; 11 General and Emergency Surgery, NHS Lothian, Edinburgh, UK; 12 Division of Trauma and Acute Care Surgery, Fundación Valle del Lili, Cali, Colombia; 13 Department of Surgery, Hospital Angeles Lomas, Mexico, Mexico; 14 Division of Trauma Surgery, School of Medical Sciences (SMS), University of Campinas (Unicamp), Campinas, Brazil; 15 General and Trauma Surgery, Addenbrooke’s Hospital, Cambridge University Hospitals NHS Foundation Trust, Cambridge, UK; 16 Department of General Surgery, University of Insubria, University Hospital of Varese, ASST Sette Laghi, Laghi, Regione Lombardia Italy; 17 General, Acute Care, Abdominal Wall Reconstruction, and Trauma Surgery, Foothills Medical Centre, Calgary, Alberta Canada; 18 Department of Surgery, College of Medicine and Health Sciences, UAE University, Al-Ain, United Arab Emirates; 19 Department of Surgery and Obstetrics and Gynecology, University of Buea, Buea, Cameroon; 20 General Surgery Department, Meihlati Hospital, Helsinki, Finland; 21 General Surgery Department, Mozir City Hospital, Mozir, Belarus; 22 General Surgery Department, Medical University, University Hospital St George, Plovdiv, Bulgaria; 23 Emergency and Trauma Surgery, Maggiore Hospital, Parma, Italy; 24 Riverside University Health System, CECORC Research Center, and Loma Linda University, Loma Linda, USA; 25 General, Emergency and Trauma Surgery Department, Bufalini Hospital, Cesena, Italy; 26 Neuro ICU, Papa Giovanni XXIII Hospital, Bergamo, Italy

**Keywords:** Coronavirus, COVID-19, Epidemia, Pademia, Mass casualties, Management, Resource, Criticalities, WSES

## Abstract

Since December 2019, the world is potentially facing one of the most difficult infectious situations of the last decades. COVID-19 epidemic warrants consideration as a mass casualty incident (MCI) of the highest nature. An optimal MCI/disaster management should consider all four phases of the so-called disaster cycle: mitigation, planning, response, and recovery. COVID-19 outbreak has demonstrated the worldwide unpreparedness to face a global MCI.

This present paper thus represents a call for action to solicitate governments and the Global Community to actively start effective plans to promote and improve MCI management preparedness in general, and with an obvious current focus on COVID-19.

“Have I missed the mark, or, like true archer, do I strike my quarry? Or am I prophet of lies, a babbler from door to door?” (Cassandra in “Agamemnon,” Aeschylus)

“Things are now as they are;

they will be fulfilled in what is fated;

neither burnt sacrifice nor libation

of offerings without fire

will soothe intense anger away.”

(“Agamemnon,” Aeschylus)

## Background

Since December 2019, the world is potentially facing one of the most difficult infectious situations of the last decades [1]. Hundreds of thousands of patients are suffering from COVID-19 infection with millions more at risk, who are presenting to the attention of the medical personnel inside and outside the hospitals. In some locations, almost 10% of cases have presented with severe respiratory impairment necessitating of intensive care. Despite intensive life support, however, many of the sick have died of this new respiratory viral infection. Beyond fatalities, a greater percentage necessitated admission to hospitals for diagnosis and treatment. Sanitary systems of different parts of the world are in great troubles, and some of them are at risk of collapsing under this infectious emergency due to discrepancies between system resilience and an overwhelming number of patients requiring attention.

## Main text

The American College of Emergency Physicians (ACEP) has defined a disaster or a mass casualty incident (MCI) as occurring “when the destructive effects of natural or man-made forces overwhelm the ability of a given area or community to meet the demand for health care” [3].

The main features of a disaster/MCI are as follows:

- It interrupts the normal functioning of a community.
- It exceeds the coping mechanisms (capacity) of the community.
- External assistance is often needed to return to normal functioning of a community [4].

Generally, an MCI conjures up imagery linked to a scene of catastrophic and impressive events either natural or man-made. This typically involves patients who are severely injured, bleeding, and screaming, brought to the hospital by emergency services, relatives, or Good Samaritans simply trying to help. This is not what the COVID-19 pandemic looks like.

Patients are coming to the hospital autonomously, generally as flu-like sick patients with sometimes cough and fever or other undefined often mild respiratory symptoms.

This situation generated some confusion and underestimation of the real weight of the problem, in the very first days. However, it is becoming clearer that the dimension of the issue may potentially be catastrophic if not correctly managed. Thus, the COVID-19 epidemic warrants consideration as a MCI of the highest nature.

Principles of MCI management present fundamental rules to be followed in order to face something that by definition is almost unmanageable [2–4].

MCIs are classified by levels, based on the number of potential victims generated [5] by

the event:

- *Level 1*: 1–10 potential victims
- *Level 2*: 11–30 potential victims
- *Level 3*: 31–50 potential victims
- *Level 4*: 51–200 potential victims
- *Level 5*: more than 200 victims
- *Level 6*: long-term operational period(s)
- The authors however propose that the worst-case scenario for COVID-19 exponentially exceeds any level previously conceptualized and that a level beyond 6 might be considered.

Alternatively, MCI can be classified even considering the entity of the response [4]—in terms

of resources—required to face them:

- *Level I:* requires local emergency response personnel and organizations to contain and deal effectively with the disaster and its aftermath.
- *Level II:* requires regional efforts and mutual aid from surrounding communities.
- *Level III:* is of such a magnitude that local and regional assets are overwhelmed, requiring national assistance.
- *Level IV:* sometimes included in level III; this MCI is of such magnitude that it requires international assistance and resources.
- Again, the authors however propose that the worst-case scenario for COVID-19 would require resources that may also exceed the capabilities of international assistance and resources, such that global cooperation becomes practically impossible. Even the wealthiest most altruistic countries may face their own MCI situations and be unable to provide the basic necessities to their own citizens.

Regardless of the classification systems of the event, some cornerstones of management exist and have been precisely defined based on several previous MCI events.

An optimal MCI/disaster management should consider all four phases of the so-called disaster cycle: mitigation, planning, response, and recovery [3]. Particular attention on certain aspects of the cycle must be given in order to adapt the action to the MCI faced. On the other hand, the unbalanced attention posed on only one aspect may increase the harmful impact of events.

These four phases are as follows:

1. *Mitigation:* Some of the devastating effects of MCI may be reduced before the event. In fact, useful measures may be posed in action in the affected region before the event happening, in all those events that can be preventable or are realistically announced (i.e., economic and political attempt to mitigate MCI effect local, regional, and national hospital and infrastructure reorganization, activities and personnel redistribution, material supplying, patient and people advising).
2. *Planning:* A realistic disaster plans involve exercise, practice, and continuous revision. In the impossibility to plan for all contingencies, plans must be relatively general and expandable but must be present. Mutual aid agreements or contracts among existing area associations, institutions, and nations (in a wider context of international entities, i.e., unions or federations) must be established before an actual event, in order to plan and optimize available resources as well as planning for funding and reimbursement. Most medical entities have not anticipated this pandemic, and therefore, planning must begin immediately and the response escalated according to the changing levels of demand/compromise.
3. *Response:* This phase is generally considered the most important one, but effective and coordinated response greatly depends on the other three phases. The main aspects of the response phase may be summarized as follows:
  *–– Activation, notification, and initial response:* Organizations and personnel involved in response and the potentially affected populations should be notified through effective and trust worth communication channels without generating panic and mass unreasonable and risky behaviors.
  As well, the different structures must be advised in time and in an appropriate manner to allow them to allocate the necessary resources and to free the necessary beds and rooms to host the sick patients. All these things must be prepared as much as possible before the first wave of sick patients’ arrival.
  –– *Organization of command and scene assessment:* Establishing a command structure is one of the most crucial steps. This must be prearranged and assembled almost immediately, as well as communication nets established. The Incident Command System (ICS) is the only that is in charge to direct and organize the different actions in the affected regions. All different regions or local directors are not allowed to manage the event differently from what defined by the ICS.
  –– *Search and rescue*
  –– *Extrication, triage, stabilization, and transport:* Triage involves providing the most efficient aid to as many as possible and prioritizes victims’ treatment, transport, and/or transfer. Many variables influence the manner in which patients are triaged, transported, and treated: kind of incident, victim number, resources available, infrastructure capability, and the disaster context. Errors in triaging lead to worse outcomes. Health care providers must be familiar with MCI triage concept and trained in performing it. Transport must equitably distribute victims to capable receiving facilities.
  On the other side, the different structures must be familiar with the necessity of resource allocation during MCI which are deeply different from the normal activity. Operating principles for those charged with performing triage must be established a priori, agreed upon by the health authorities and society, and especially transparent to the general public. In a true MCI, the greatest good must be provided to the greatest number of patients. When there is a 10 to 1 ratio of those in need of life-supporting technologies versus available infrastructure, the sickest may unfortunately be denied care and moved into an expectant category.
  –– *Definitive scene management*
  *Unique aspect of the pandemic—*Surgeons and some ultra-specialistic branches of health system provide a unique service that cannot be performed by other medical disciplines. Therefore, all of them must be secured in a protected environment, minimizing risks of acquiring the disease because critically ill and injured patients will continue to need emergent care.

4. *Recovery:* This last phase is crucial for the affected community. Management of the involved personnel is very important during this phase for critical stress debriefing. Debriefing may teach valuable lessons. It is crucial to obtain as much information as possible from all actively involved participants. If complete and objective criticality analysis is not performed, improvements in future responses are impossible [3].

Even without a more in-depth analysis of MCI management is self-evident that management of the COVID-19 outbreak has demonstrated the worldwide unpreparedness to face a global MCI especially if “unconventional.” According to the stratification and grading systems, this COVID-19 event is beyond even the most serious and demanding of the levels (i.e., level 6 or level IV). The number of expected victims in fact is enormous, and the resources necessitated are near beyond imagination, never mind current infrastructure.

This means that the effort and the attention must be at maximum level. No discount could be done to not pay too high price in term of human lives, or in the worst scenario of system collapse. Not only the sanitary and organizational system is affected, but all the different national economic, political, and infrastructural sides are in challenge. The absolute anomaly of this MCI is the velocity and the “unconventional” diffusion and presentation. Given the consequences which are almost unimaginable, management may better default to established principles fully ascribable to an MCI, with responses managed accordingly. Simply put, it is impossible for the world to face such a potentially catastrophic scenario without a well-organized and prepared system.

However, an analysis of the actual characteristics features of the early responses may provoke many criticisms:

- Lack of effective presence of national and international agencies for disaster management and of international communication, collaboration, and support. In reality, international organizations in charge in disease prevention and control have published quickly protocols and guidelines (sometimes conflicting with each other as in the case of protective masks for health workers), but they have been implemented very slowly at national level. The lack of coordination of the activities between and within the international and national levels was evident in the first phase.
- Lack of mitigation phase during which many strategies may have been posed into action to reduce the real impact of the infection on the different systems. Taking into account the evolution of Chinese cases and the progressive spread within the world, there would have been time to organize a mitigation phase.
- Lack of an effective action planning at international and national level. The action plans have progressively followed. Probably, a well-structured action plan aimed at containing a potential disaster would have created fewer difficulties in managing COVID-19.
- Lack of national central directed strategies to prevent infection transmission and to timely isolate the more severely affected regions. The epidemic initially spread to those countries that are probably less virtuous in infection prevention and control. In these countries, the problem was handled by policymakers and technicians who did not perceive the real potential of this emergency.
- Lack of trust worth and objective information to the potential affected people given from the very beginning. The information was immediately conflicting, and social networks giving voice to everyone, which complicated the information transmission chain.
- Lack of a pre-defined disaster management plan including the need to prevent infected people from entering the emergency departments and to place the triage systems outside the emergency departments from the beginning of the infection outbreak. An infective emergency scenario, such as the present one, can repeat itself. In the coming months, the WSES will try to organize courses which will teach the concept of MCI to infectious disease emergencies, involving all interested professions. In fact, WSES has among its members medical and health professionals from different countries of the whole world who are monitoring and helping to develop actions to prevent and control this pandemic event. As most of them attend emergency areas in hospitals, they are professionals who are also at risk of contracting the disease, and at that moment, no matter the specialty or qualification of each one, they must all be united and focused on fighting this disease.
- The need to monitor the data on number of patients with related diagnoses before the first case of a patient with this type of disease is identified. If there are anomalies, then specific actions should be taken to identify the cause of this variation. It is essential to avoid any delays in identifying patient 0 or the first patients with this disease.
- Thousands of health care workers have been infected amid the ongoing coronavirus outbreak, a sign of the immensely difficult working conditions for doctors, nurses, and health care workers in general. They should be instead among those best protected. The infections, along with the deaths of several doctors in China, underscore the deeply challenging, chaotic environment that health care workers face with when toiling on the front lines of an epidemic outbreak. They face long hours, changing protocols, potential medical supply shortages, and risks to their own personal health and that of their loved ones. In every mass casualty event, the health care workers who go to the forefront are the main actors. The lack of national and international action plans forced health workers to work in a situation of extreme unsafety.

While greatly concerning, these findings should not be surprising. In previous surveys by the World Society of Emergency Surgery (WSES) conducted in 2015 [6] and the report of the National Association of Emergency Medical Technicians (NAEMT) published in 2017 [7], the most medical and disaster response systems are actually unprepared to face MCI.

Despite these reports and the unfortunate but demonstrative experiences of many countries, global preparedness toward MCI is still not institutionalized by proper management plans, promoted by governments, and nor are health care personnel sufficiently trained for the challenges likely to come [8–10].

COVID-19 clearly has demonstrated a double fold message:

- That current systems are vulnerable and extremely weak in this era of rapid global travel in containing an MCI that is paradoxically moving not so fast and with not immediately life threating sick patients.
- As United Nations High Commissioner for Human Rights Michelle Bachelet commented “Human rights need to be front and center in response (...) to effectively combat the outbreak means ensuring everyone has access to treatment, and is not denied health care because they cannot pay for it or because of stigma (…)” [11].

As different social groups and local communities have different and diverse understandings of scientific knowledge [12], the key to addressing such a global public health outbreak could be adopting a uniform, evidence-based approach [13] that puts public risk perception at the center. Communities and individuals need to work together along with academia and policymakers. Sensitization and prevention measures are necessary. However, there cannot be a one size fits all approach for all countries. The expected health, security, and economic benefits of measures need to be weighed against any expected health, security, and economic costs, as well as any “moral” costs intrinsic to coercion and compulsion [14].

## Conclusion

This present paper thus represents a call for action to solicitate governments and the Global Community to actively start effective plans to promote and improve MCI management preparedness in general, and with an obvious current focus on COVID-19. Global Health cannot continue to proceed considering mass medical emergencies extraordinary situations when they are part of the natural course of things. As natural parts of the common life, Global Health must be prepared to face them at the best of our possibilities. Without considering even the economic and political drawbacks, if actions will not be promoted, with the onset of the next MCI, it can be prevented that the number of affected and dead people will be higher and higher.

## Data Availability

Not applicable

## Abbreviations

ACEP: American College of Emergency Physicians
ICS: Incident Command System
MCI: Mass casualty incident
NAEMT: National Association of Emergency Medical Technicians
WSES: World Society of Emergency Surgery
